# Inflammatory Response of Primary Cultured Bovine Mammary Epithelial Cells to *Staphylococcus aureus* Extracellular Vesicles

**DOI:** 10.3390/biology11030415

**Published:** 2022-03-09

**Authors:** Mara D. Saenz-de-Juano, Giulia Silvestrelli, Andres Weber, Christian Röhrig, Mathias Schmelcher, Susanne E. Ulbrich

**Affiliations:** 1 Animal Physiology, Institute of Agricultural Sciences, ETH Zurich, 8092 Zurich, Switzerland; mara.saenz@usys.ethz.ch (M.D.S.-d.-J.); giulia.silvestrelli@usys.ethz.ch (G.S.); andres.weber@llv.li (A.W.); 2 Institute of Food, Nutrition and Health, ETH Zurich, 8092 Zurich, Switzerland; c.roehrig@micreos.com (C.R.); mathias.schmelcher@hest.ethz.ch (M.S.)

**Keywords:** *Staphylococcus aureus*, extracellular vesicles, bovine mammary epithelial cells, gene expression

## Abstract

**Simple Summary:**

Mastitis, the inflammation of the mammary gland, is one of the most common and costly diseases worldwide, and *Staphylococcus aureus* (*S. aureus*) is among the most prevalent microorganisms that cause it. To obtain new insights into *S. aureus* mammary gland infections, we have isolated *S. aureus* extracellular vesicles to challenge in vitro primary bovine mammary epithelial cells. Despite the toxic content of the vesicles, we observed only a minor pro-inflammatory response. The latter can contribute to the explanation of how *S. aureus* evades mammary epithelial defence mechanisms and successfully colonizes the mammary gland.

**Abstract:**

In dairy cows, *Staphylococcus aureus* (*S. aureus*) is among the most prevalent microorganisms worldwide, causing mastitis, an inflammation of the mammary gland. Production of extracellular vesicles (EVs) is a common feature of *S. aureus* strains, which contributes to its pathogenesis by delivering bacterial effector molecules to host cells. In the current study, we evaluated the differences between five *S. aureus* mastitis isolates regarding their EV production. We found that different mastitis-related *S. aureus* strains differ in their behaviour of shedding EVs, with M5512VL producing the largest amount of EVs containing alpha-haemolysin, a strong cytotoxic agent. We stimulated primary cultured bovine mammary epithelial cells (pbMECs) with EVs from the *S. aureus* strain M5512VL. After 24 h of incubation, we observed a moderate increase in gene expression of tumour necrosis factor-alpha (*TNF-α*) but, surprisingly, a lack of an associated pronounced pro-inflammatory response. Our results contribute to understanding the damaging nature of *S. aureus* in its capacity to effectively affect mammary epithelial cells.

## 1. Introduction

Mastitis, the inflammation of the mammary gland, is one of the most costly diseases in dairy farming worldwide [1]. Apart from the reduction in milk yield and quality, mastitis involves other costs such as discarded milk due to antibiotic treatment, culling or veterinary treatments. Although many bacteria can cause mastitis, there are only a few that are particularly prevalent and generate a real issue for dairy farms [2]. This is the case of the Gram-positive bacterium *Staphylococcus aureus* [2,3]. *S. aureus* triggers a moderate response (subclinical mastitis) that remains asymptomatic, but can easily be transmitted to other cows in the herd during routine milking and ultimately result in chronic or life-long disease [1,4].

One of the most problematic abilities of *S. aureus* is to reside intracellularly within mammary epithelial cells (MECs) without activating the innate immune response of the infected host [2]. It has been observed that subclinical mastitis can be experimentally induced by introducing a low number of bacteria (approximately 100 colony-forming units) through the teat, demonstrating that the natural defence mechanisms of the mammary gland against infection are inefficient [5,6]. It has been postulated that *S. aureus* infections are associated with a failure to activate pattern recognition receptor (PRR)-signalling cascades, which impairs neutrophil recruitment and enables *S. aureus* to be established in the mammary gland [7]. It is known that MECs can efficiently sense and react against isolated pathogen-associated molecular patterns (PAMPs) of *S. aureus* such as lipoteichoic acid (LTA), but they fail to recognize intact *S. aureus* despite being readily invaded [3].

Vesicular transport is a universal phenomenon used by many different cell types, including microorganisms. The cargo of extracellular vesicles (EVs) of microorganisms contains nucleic acids, toxins, lipoproteins and enzymes. They play important roles in microbial physiology and pathogenesis (reviewed by [8,9]). Studies investigating *S. aureus* EVs have usually focused on *S. aureus* human clinical strains [10,11,12,13,14,15,16,17,18]. Nevertheless, *S. aureus* EVs have been also successfully isolated from several mastitis-related strains: USA300 [19], N305 (ATCC29740 [20]), RF122, O11 and O46 [21]. Secreted *S. aureus* EVs are bi-layered membranous structures with diameters ranging from 20 to 300 nm [16,17,21]. Proteome analyses of EVs of different *S. aureus* strains revealed a content highly enriched in specific proteins responsible for transport, virulence or pathological functions, and suggested a specific sorting mechanism for the excretion of EVs [16,20,22]. Importantly, EVs derived from *S. aureus* can also contain a significant amount antibiotic-resistance proteins, which enables other ampicillin-susceptible Gram-negative and Gram-positive bacteria to survive in the presence of ampicillin (reviewed by [22]).

It has been shown in vivo that *S. aureus* EVs can induce apoptosis of lung cells [15] and clinical signs of arthritis in knees [17], and can cause a local inflammatory reaction in the mouse mammary gland [21]. In vitro, *S. aureus* EVs induce a dose-dependent transcriptional upregulation of interleukin 1 beta (*IL-1β*), interleukin 8 (*IL-8*), tumour necrosis factor-alpha (*TNF-α*) and defensin β-1 (DEFβ1) in the bovine mammary epithelial cell line PS [20]. However, cell lines tend to lose the phenotypes of the original tissue [23,24], and it is known that MAC-T responds weaker than pbMEC to immune stimulations, probably due to some degree of dedifferentiation and an attenuated TLR-signalling [25].

Finally, since *S. aureus* EVs contain many bacterial proteins (including cell surface proteins and toxins) and do not require adjuvants to elicit an effective adaptive immune response, they have been postulated as an innovative strategy in vaccine development against *S. aureus* infections [9,10,19,26].

To gain new insights into the role of *S. aureus* EVs in bovine mammary gland infection and associated strain-dependent differences, we (i) analysed particle concentration, morphology and size of EVs from five *S. aureus* mastitis strains (USA300, N305, M5712, Mastidis, M5512VL) and (ii) evaluated the effect of *S. aureus* M5512VL-derived EVs in vitro in primary bovine mammary epithelial cells (pbMECs) on the transcriptional inflammatory response.

## 2. Materials and Methods

### 2.1. S. aureus Culture

Five different *S. aureus* strains from the Laboratory of Food Microbiology collection at ETH Zurich (Zurich, Switzerland, Table 1) were grown at 37 °C in 1 L of Luria-Bertani (LB) medium (10 g/L tryptone, 5 g/L yeast extract, 8 g/L NaCl (pH 7.4)) for 2–3 h and 150 rpm shaking. When the optical density at 600 nm (OD_600_) of 1.0 was reached, the culture was cooled down at 4 °C. Specifically for S. aureus, an OD600 of 1.0 as more or less 1–10 × 10^9^ CFU/mL has been reported [27]. Afterwards, the culture was centrifuged at 6000× *g* for 15 min, and the supernatant was filtered with a 0.22 µm filter and stored at 4 °C upon further ultracentrifugation. Then, the bacteria were heat-killed at 60 °C for 30 min and divided into 2 mL aliquots before freezing.

### 2.2. EVs Isolation from S. aureus Cultures

All procedures before and after ultracentrifugation were performed under the sterile hood. Part of the filtered medium (150 mL) was loaded in four 38.5 mL Beckman UltraClear tubes (Beckman Coulter Corp., Brea, CA, USA) and centrifuged in a Beckman Coulter Optima XE-90 (Beckman Coulter Corp., Brea, CA, USA) in a fixed-angle rotor 50.2 Ti (Beckman Coulter Corp., Brea, CA, USA) at 150,000× *g* for 2 h at 4 °C. After ultracentrifugation, the supernatant was discarded, and the pellet in each tube was resuspended in 20 µL of PBS (Thermo Fisher Scientific, Waltham, MA, USA). The pellets containing the same media from a single bacterial culture were pooled together and stored at −80 °C.

For the pbMECs challenge, an additional 500 mL of filtered culture was ultracentrifuge to obtain enough *S. aureus* EVs to reach 10 µg for each stimulation.

The sterility of *S. aureus* EVs was tested by adding 10 µL of *S. aureus* M5512VL EV pellet to an LB agar plate and incubating 24 h at 37 °C (Supplementary Figure S1).

### 2.3. Protein Concentration Analysis

A total of 50 µL of EVs sample was mixed with 10 µL of radioimmunoprecipitation assay (RIPA) buffer containing 1% antiprotease and 1% ethylenediaminetetraacetic acid (EDTA). Protein concentration was measured using the Pierce™ BCA Protein Assay Kit (Thermo Fisher Scientific, Waltham, MA, USA) coupled with the Nanodrop 2000 (Thermo Fisher Scientific) according to the manufacturer’s instructions.

### 2.4. Transmission Electron Microscopy (TEM)

Visualization of EVs was performed through the Scientific Center for Optical and Electron Microscopy (ScopEM) service of ETH Zurich. Briefly, three microliters of the vortexed dispersion of EVs was placed on glow discharged carbon-coated grids (Quantifoil, D) for 1 min. Negative contrast staining was done in 2% sodium phosphotungstate pH 7.2 for 1 s, followed by a second step for 15 s. Excess moisture was drained with filter paper, and the imaging of the air-dried grids was done in a TEM Morgagni 268 (Thermo Fisher Scientific, Waltham, MA, USA) operating at 100 kV.

### 2.5. Tuneable Resistive Pulse Sensing (TRPS)

Particle concentration and size distribution were measured using the qNano Gold system (Izon Science Ltd., Christchurch, New Zealand) and an NP150 Nanopore. CPC100 beads were used as the calibration standard. Particles were measured using 46.0 mm stretch with a voltage of 1.4 V and a pressure of 8.12 mbar. The number of particles analysed per sample was at least 500. The blockade magnitude of the calibration particles was above 0.2 nA. Data were processed using the Izon Control Suite software version 3.3.

### 2.6. Western Blot

Samples (5 µg of protein) mixed with Laemmli Buffer (Bio-Rad Laboratories, Inc., Hercules, CA, USA) were loaded into a 12-well Mini-PROTEAN^®^ TGX Stain-Free™ Precast Gel (Bio-Rad). The stain-free gel was UV activated using the ChemiDocTM MP (Bio-Rad), and proteins were transferred onto a 0.2 µm PVDF trans-blot turbo transfer pack (Bio-Rad Laboratories, USA) using the Turbotransfer (Bio-Rad) and Bio-Rad Mixed Mw protocol. Immediately, the membrane was blocked with TBST (0.05% Tween 20) with 5% skim milk powder at room temperature for 1 h. Afterwards, the membrane was incubated overnight with the primary antibody mouse anti-α-haemolysin (Hla) (ab190467, Abcam, Cambridge, UK). To evaluate the unspecific 50 kDa band, two more membranes were incubated with only the blocking buffer or with the CD81 primary antibody (sc-166029, Santa Cruz Biotechnology, Dallas, TX, USA). On the next day, the three membranes were washed and incubated with the secondary antibody goat anti-mouse IgG-HRP (Santa Cruz Biotechnology, sc2005, 1:10,000). To visualise the ladder, Precision Protein Strep Tactin-HRP was added as well (1:10,000, Bio-Rad). Finally, Clarity^TM^ ECL substrate (Bio-Rad) was loaded onto the membrane and bands were visualized with ChemiDocTM MP. 

### 2.7. Primary Bovine Mammary Epithelial Cells (pbMECs) Culture

Primary cells were extracted from mammary gland parenchymal tissues of 6 lactating cows collected at a local slaughterhouse. Tissues pieces of 10 g were washed in ethanol 70% and Phosphate Buffer Saline (PBS) containing antibiotics and antimycotics, cut into smaller pieces (approximately 1 mm^3^) and digested for 3 h at 37 °C with gentle agitation, in a digestive mix containing collagenase IV 0.5 mg/mL, dispase II 0.5 mg/mL, insulin 5 µg /mL, gentamicin, penicillin and streptomycin in Hank’s Balanced Salt Solution (HBBS). After several washing steps, cells were resuspended in Dulbecco’s modified Eagle medium (DMEM/F12) (Thermo-Fisher Scientific, Waltham, MA, USA) supplemented with Gentamicin (Sigma-Aldrich Corp., St. Louis, MO, USA) 1:1000 and amphotericin B (AmpB, Thermo-Fisher, Waltham, MA, USA) 1:100 and 10% foetal bovine serum (FBS). The cells were counted and checked for viability using Trypan blue staining (Sigma-Aldrich Corp., St. Louis, MO, USA). Cells were cultured on a six-well plate at 37 °C, 5% CO_2_ in 95% humidified air, with a density of approximately 600,000 cells/well.

### 2.8. Experimental Challenge of pbMECs

Six cultures of primary bMECs were stimulated with heat-killed *S. aureus* M5512VL, LTA (10 µg, L2515, Sigma-Aldrich Corp., St. Louis, MO, USA) and *S. aureus* EVs M5512VL (10 µg) in three different sessions (two cultures per session). The EVs belonged to the same M5512VL bacterial culture, and the dose corresponding to the quantity of protein similarly to what was previously published [17]. All of the applied treatments were diluted in 1.5 mL DMEM/F12 medium supplemented with Gentamicin and AmpB, except for the control condition (Ctr), in which only medium with Gentamicin and AmpB was added. After 3 or 24 h of incubation, cells were washed twice with PBS, and 300 µL of Trizol™ (Thermo-Fisher Scientific, Waltham, MA, USA) was added per well to detach the cells. Afterwards, the mixture was transferred to a tube and immediately frozen at −80 °C until further analysis.

### 2.9. RNA Extraction and Reverse Transcription

RNA from pbMECs was extracted using phenol-chloroform. Briefly, 60 µL of chloroform was added to the Trizol™ reagent. After 7 min of incubation at room temperature (RT), tubes were centrifuged for 15 min at 4 °C at 12,000× *g*. Then, the upper phase was transferred to a new tube containing isopropanol. After 7 min of incubation at RT, RNA was precipitated by centrifuging tubes at 12,000× *g* for 10 min at 4 °C. Finally, RNA pellets were washed with two rounds of ethanol 75% and resuspended in 10 µL of RNase-free water. To avoid DNA contamination, a DNase treatment step was included (Thermo-Fisher Scientific, Waltham, MA, USA). RNA concentration and quality were measured immediately using a Nanodrop 2000 (Thermo Fisher Scientific, Waltham, MA, USA), and samples were stored at −80 °C. Reverse transcription was performed using 500 ng of RNA and the Promega GoScript (Promega Corp, Madison, WI, USA).

### 2.10. Quantitative PCR

Quantitative PCR (qPCR) was performed using 1 µL of cDNA, 10.0 µL KAPA SYBR^®^ FAST (Sigma-Aldrich Corp., St. Louis, MO, USA), 0.8 µL forward and reverse primer at a concentration of 10 µM (Table S1), respectively, and 7.4 µL RNase-free water per sample, respectively. As calibration standard, 1 µL of pooled cDNA was run with the same conditions as the other samples. For each gene, a non-target control (NTC) sample was included. All samples and controls were run in duplicates in a CFX ConnectTM (Bio-Rad Laboratories Inc., Hercules, CA, USA). The qPCR protocol included an initial step of 50 °C (2 min), followed by 95 °C (10 min) and 42 cycles of 95 °C (15 s) and 60 °C (30 s). After real-time PCR, a melting curve analysis was performed by slowly increasing the temperature from 65 °C to 95 °C, with continuous recording of changes in fluorescent emission intensity. Amplification products were confirmed by GelRed-stained 1.5% agarose gel electrophoresis. The formula used to calculate the relative expression in pbMEC was ΔCt = Ct[reference gene] − Ct[target gene] + 15. As a reference, we employed the geometric average of beta-actin (*ACTB*) and glyceraldehyde-3-phosphate dehydrogenase (*GAPDH*).

### 2.11. Statistical Analysis

Statistical analyses were performed in GraphPad Prism 8 (GraphPad Software, San Diego, CA, USA). Shapiro–Wilk test was applied to every data set to test for normal distribution. Then, a paired T-test was applied for all groups against the control. Significant differences were considered when *p* ≤ 0.05. Gene expression data are presented as mean ± standard deviation of 6 biological replicates of pbMECs coming from different individuals.

## 3. Results

### 3.1. Differences in Extracellular Vesicle Isolation from Five S. aureus Mastitis Strains

**Figure 1:**
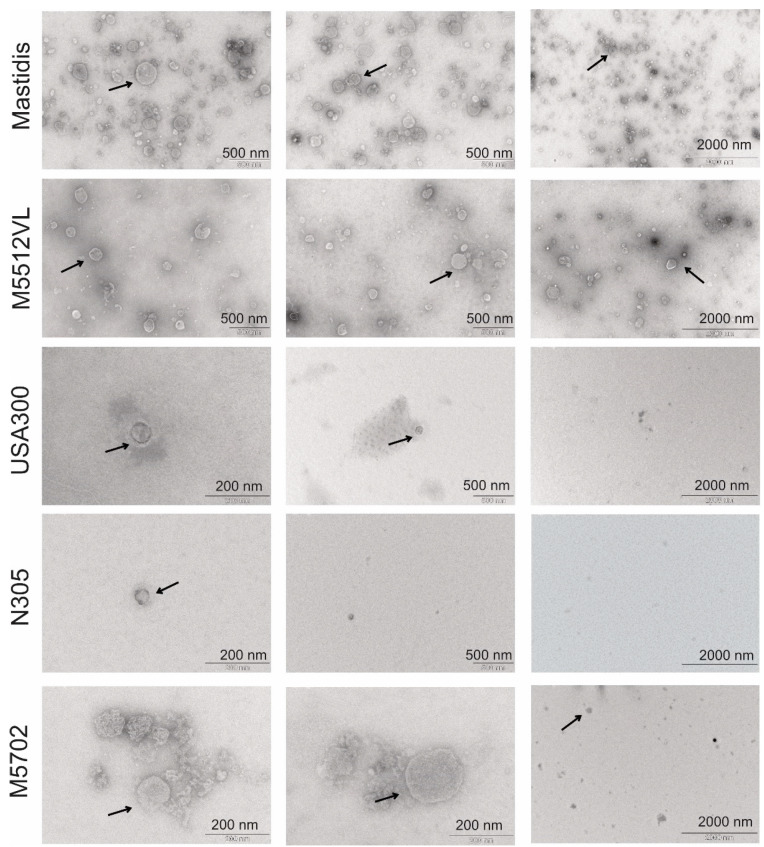
Transmission electron microscopy (TEM) observations of isolated *S. aureus* EVs for each mastitis strain Mastidis, M5512VL, USA300, N305 and M5702. Black arrows indicate EVs.

TEM demonstrated that different *S. aureus* strains secreted EVs into the media (Figure 1). Mastidis and M5512VL were the strains that produced more nano-sized vesicular structures with a typical cup shape (Supplementary Figures S2 and S3).

**Figure 2:**
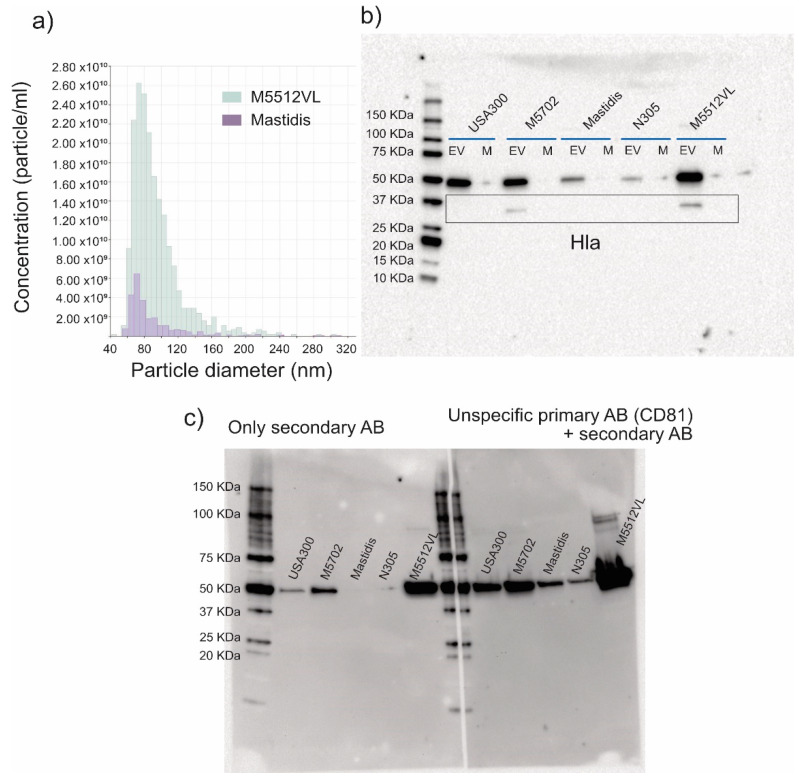
(**a**) Representative graphs of size distribution and particle concentration of *S. aureus* M5512VL EV (grey) and *S. aureus* Mastidis EV (purple), determined by tunable resistive pulse sensing technology (TRPS). (**b**) Western blot characterization for alpha-haematoxylin (Hla) using the same amount of total protein from EVs and culture media before EV isolation (M) of each *S. aureus* strain. (**c**) Negative control Western blots. The image shows an unspecific band at 50 KDa from the secondary AB. After the blocking step, the membrane was cut in two. The left part of the membrane was incubated with blocking buffer overnight and with the secondary antibody, while the right part was incubated with the unspecific primary antibody (CD81) that is not present in *S. aureus* and with the secondary antibody. Original Western blot figures are shown in Supplementary Figure S4.

These results were confirmed by TRPS analysis (Figure 2a). M5512VL was the strain with the highest concentration of vesicles, followed by Mastidis (Table 2). Despite the EVs shown in the TEM images, the particle rates from M5702, USA300 and N305 were too small (>100 particles/minute) to perform an accurate TRPS measurement. The mean and mode particle diameter were very similar between M5512VL and Mastidis (Table 2). However, despite there being no measurable particles in M5702, USA300 and N305, we could detect similar amounts of proteins as in M5512VL or Mastidis (Table 2).

### 3.2. EVs from S. aureus M5702 and M5512VL Contain Alpha-Haematoxylin (Hla)

Western blot showed that not all *S. aureus* strains produced EV with Hla, and only M5702 and M5512VL presented a band at the expected position of 36 kDa (Figure 2b). Importantly, these bands were only seen in the ultracentrifuged pellet and not in the culture media (Figure 2b). We also found a strong band at 50 kDa, likely due to the unspecific binding of the secondary antibody by staphylococcal protein A (Figure 2c).

### 3.3. Gene Expression Analysis of Stimulated pbMECs with S. aureus M5512VL EVs

**Figure 3:**
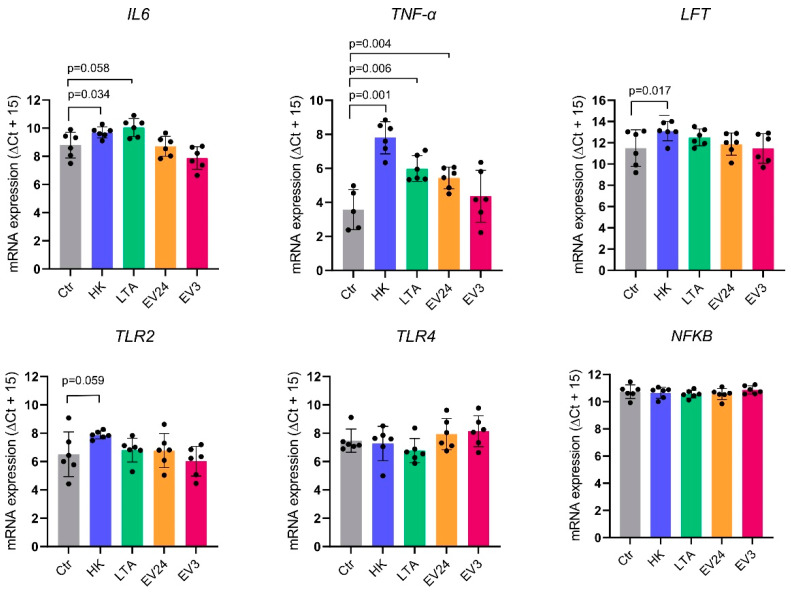
Differential expression analysis for interleukin 6 (*IL-6*), tumour necrosis factor-alpha (*TNF-α*), lactoferrin (*LTF*), toll-like receptor 2 (*TLR2*), toll-like receptor 4 (*TLR4*) and nuclear factor-kappa B (*NFκB*) of primary bovine mammary epithelial cells (pbMECs) after stimulation with PBS (Ctr), heat-killed *S. aureus* M5512VL (HK), lipoteichoic acid (LTA) for 24 h, or *S. aureus* M5512VL EVs for 24 (EV24) and 3 h (EV3) stimulation, respectively. Relative abundance values are expressed in ∆Cq, showing the mean value + SD from six biological replicates.

Primary bMECs were stimulated with *S. aureus* M5512VL EVs for 3 h (EV3) or 24 h (EV24). Heat-killed (HK) *S. aureus* M5512VL and LTA were used as a positive control after 24 h incubation. Differential expression of interleukin 6 (*IL-6*) was observed for HK and LTA but not for EV3 and EV24 (Figure 3). A significant upregulation of lactoferrin (*LTF*) and toll-like receptor 2 (*TLR2*) was also observed for HK but not for LTA or EV (Figure 3). After HK and LTA stimulation, tumour necrosis factor-alpha (*TNF-α*) had the highest upregulation (Figure 3, *p* < 0.05). Interestingly, we also detected a significant upregulation after 24 h of EV stimulation, but not after 3 h. No significant differences were detected in gene expression of nuclear factor-kappa B (*NFκB*) and toll-like receptor 4 (*TLR4*) (Figure 3).

## 4. Discussion

It is known that EV production is a common feature of *S. aureus* strains that contributes to its pathogenesis by delivering bacterial effector molecules to host cells [14]. The most common methods for *S. aureus* EVs isolation usually involve ultracentrifugation coupled to density gradients such as Optiprep [16,19,26,29] or sucrose [15,20,30]. In this study, we isolated *S. aureus* EVs similarly to what was previously published by performing ultracentrifugation after bacteria removal with a 0.22 µm filter [17,18,31,32]. A small part of the *S. aureus* secretome could have been retained, because to speed the processing time and avoid a loss of starting material, we avoided washing the resulting pellet with 1× PBS. This can contribute to the explanation of why we observed similar protein concentrations but different amounts of EVs in all pellets.

We investigated the difference in EV production between five different *S. aureus* strains related to mastitis (USA300, N305, M5712, Mastidis, M5512VL) and we characterized the isolated EVs with typical techniques used for mammalian EVs such as TEM, TRPS and Western blot [21]. TEM imaging proved that round-shaped EVs of different sizes could be successfully isolated. Mastidis and M5512VL had the highest particle concentration, with a mean diameter in the range of 90–95 nm, which has been reported as typical for *S. aureus*-derived EVs [16,21]. On the contrary, N305, USA 300 and M5702 could not be analysed by TRPS due to the low particle concentration, which we also observed in representative TEM images. Recently, Tartaglia et al. [20] observed with TRPS comparable amounts of EVs for the N305 strain to what we did for Mastidis and M5512VL. The reason for this difference regarding the amount of purified EVs could be that the previous authors used more volume as starting material than we did. Specifically, they used 1 L of filtered medium that was concentrated around 100-fold before ultracentrifugation, while we only used 150 mL of filtered medium. Additionally, the EV isolation protocol and TRPS conditions employed were also different, and these can be critical factors for the outcome. Despite Mastidis and M5512VL being the *S. aureus* strains with the highest EV secretion, these strain-specific differences in EV formation could also change under the physiological conditions found in cow udders and impact their inflammatory potential. In this sense, it has been shown recently that JE2 USA300 *S. aureus* incubated at 30 °C yielded more EVs when grown to the same optical density at 37 °C [29].

Alpha-Haemolysin (Hla), also known as α-toxin, is the best-characterized virulence factor of *S. aureus* (reviewed by [33,34]). Upon binding to the cell surface, Hla forms pores in the lipid bilayer of eukaryotic host-cells, leading to its necrotic death. Hla has been detected in more than 200 bovine mammary isolates of *S. aureus* [35] and specifically in *S. aureus* ATCC14458 EV proteome [16]. In our Western blot analysis, only M5702 and M5512VL strains showed the band for Hla. It has been published that N305 and USA300 have Hla in their genome (Gene Bank AKYW00000000 and EMBL Nucleotide Sequence Database GCA_000013465, respectively). We do not know if this is the case for Mastidis. This could explain why, despite having a high amount of EVs, the Mastidis strain did not show the presence of Hla, further strengthening the hypothesis that *S. aureus* virulence variability in bovine mastitis is highly dependent on strain-specific features [36]. Thus, heat-killed *S. aureus* M5512VL and its EVs were chosen to challenge in vitro pbMECs.

In a Western blot performed only with the secondary antibody (negative control), we detected that all EVs samples presented an unspecific band in the range of 50 kDa. We hypothesize that this band might correspond to the *S. aureus* Protein A (SpA), a specific membrane protein of *S. aureus* that binds the Fcγ domain of immunoglobulin (Ig) and cross-links the Fab domain of VH3-type B cell receptors (IgM) [37]. Proteomic analysis of *S. aureus* EVs suggested that many biologically active bacterial proteins are packaged onto *S. aureus* EVs [16], and specifically, SpA was previously identified in the human isolate *S. aureus* 06ST1048 EV [15]. As the amount of total EV protein used in the Western blots was adjusted to the same concentration, differences in the intensity of the detected bands are likely due to differences in the abundance of SpA protein.

Since MECs contribute to more than 70% of all cells from the lactating udder, they might be responsible for directing the immune reaction within the udder early after infection [25]. In this regard, MECs are highly relevant for both sentinels as well as effector functions of immune defence in the udder [25]. When MECs sense the pathogens through their Pattern-Recognition Receptors (PRRs), they secrete antimicrobial molecules and cytokines to recruit circulating immune cells, especially lymphocytes and neutrophils [38]. In addition, MECs rather than immune cells can also fine-tune their responses to different invading pathogens by producing different cytokine sets [7]. In this study, we evaluated whether *S. aureus* M5512VL EVs induce an in vitro immune response in MECs comparable to that of both heat-killed *S. aureus* and LTA. We used primary cultures of bMECs because they reflect better than the cell lines the in vivo infected udder, and in vitro cultured pbMECs have a pathogen-specific activation of innate immune mechanisms [25]. Moreover, primary cell isolates inherently reflect the individual variability between donors [25]. The transcript abundance of *IL-6*, *TLR2*, NF-κB, *TLR4,* and *LTF* genes have not been reported yet in pbMECs after *S. aureus* EVs stimulation. We also analysed the gene expression of *TNF-α*, which was detected as upregulated in the cell line PS when stimulated with *S. aureus* N305 EVs [20].

Stimulation of pbMECs with heat-killed *S. aureus* M5512VL significantly upregulated the expression of *IL-6*, *LTF*, *TLR2,* and *TNF-α*, confirming that our in vitro pbMECs conserved the immune mechanisms to efficiently respond to *S. aureus* infection. As expected, the expression of neither NF-κB nor *TLR4* was upregulated by any *S. aureus* M5512VL endotoxins [25]. It is known that neither *S. aureus* infection nor in vitro stimulation lead to an activation of the NF-κB factor complex, explaining in part the low-intensity immune response of MECs compared to other pathogens such as *Escherichia coli* [39].

Challenging pbMECs with *S. aureus* M5512VL EVs induced a significant upregulation of *TNF-α* after 24 h. *TNF-α* is a pro-inflammatory cytokine and a key component of the innate immune response of MECs responsible for neutrophil recruitment [7]. The lower increase in *TNF-α* gene expression after EV compared to HK and LTA treatments might be explained by the ability of MECs to modulate *TNF-α* secretion depending on the load of the pathogen [40]. The upregulation of *TNF-α* due to *S. aureus* EVs was previously observed when stimulating mammary PS cell lines with *S. aureus* EVs [20], but also in mouse-bone-marrow-derived dendritic cells, macrophages and splenocytes [10,17,41]. Further studies should evaluate whether the change in gene expression is reflected in protein-level differences.

The fact that *S. aureus* EVs harbour a broad spectrum of different content, including toxins, proteins involved in antibiotic resistance and other virulence factors, but induce a moderate *TNF-α* upregulation in MECs, might explain a mechanism used by *S. aureus* to harm the host cells while evading the host’s innate immune system [8,20]. Taking this into account, our results suggest that *S. aureus* might use EVs to affect target cells while evading to activate the immune response.

In conclusion, our results showed that different *S. aureus* mastitis strains produce different amounts of EVs. Moreover, our gene expression analysis elucidated that *S. aureus* M5512VL EVs stimulation neither upregulated *IL-6* nor *TLR2* as heat-killed *S. aureus* M5512VL does. Nevertheless, a moderate but significant upregulation of *TNF-α* was observed when stimulating with EVs. Our observations provide more information about the early phase of *S. aureus* infection. Herein, EVs containing virulence factors might be secreted by live pathogens to harm the cells without being recognized as a pathogenic agent, until their accumulation in the alveolar fluid is high enough to significantly alter the MEC immune responsiveness.

## Figures and Tables

**Table 1:** Bacterial strains used in the current study.

*S. aureus* Strain	Source
USA300 JE2	NR-46543, Network on Antimicrobial Resistance in *Staphylococcus aureus* (NARSA) [28]
M5702	Hans Ulrich Graber, Agroscope, Liebefeld, Switzerland
Mastidis	Roger Stephan, University of Zurich, Zurich, Switzerland
Newbould 305 (N305)	ATCC 29740 (American Type Culture Collection, Manassas, VA, USA)
M5512VL	Hans Ulrich Graber, Agroscope, Liebefeld, Switzerland

**Table 2:** Particle concentration, mean and mode particle diameter and protein concentration were measured in each pellet of *S. aureus* EVs.

*S. aureus* Strain	Particle Concentration (Particles/mL)	Mean Particle Diameter (nm)	Mode Particle Diameter (nm)	Protein Concentration (µg/µL)
USA300 JE2	n.d	n.d	n.d	1.1
M5702	n.d	n.d	n.d	1.3
Mastidis	1.13 × 10^10^	90 ± 34.9	71 ± 3.4	1.2
Newbould 305 (N305)	n.d	n.d	n.d	1.6
M5512VL	1.93 × 10^11^	95 ± 32.5	74 ± 3.0	1.309

n.d = not detected.

## Data Availability

Not applicable.

## Supplementary Materials

The following are available online at https://www.mdpi.com/article/10.3390/biology11030415/s1, Table S1: Sequences of forward and reverse primers used for quantitative real-time PCR (qPCR) analysis. Figure S1: sterility test of *S. aureus* M5512VL. Figure S2: Transmission electron microscopy (TEM) observations of isolated EVs from *S. aureus* Mastidis. Figure S3: TEM observations of isolated EVs from *S. aureus* M5512VL. Figure S4: Original Westren blot images.
