# Supplementary material for: Inflammatory Response of Primary Cultured Bovine Mammary Epithelial Cells to Staphylococcus aureus Extracellular Vesicles
Source: Biology (Basel). 2022 Mar 9;11(3):415. doi: 10.3390/biology11030415 (PMC8944978; doi:10.3390/biology11030415)
Supplement: Supplementary file 1 [file biology-11-00415-s001.zip › biology-1593024-supplementary(1).pdf]

biology MDPI

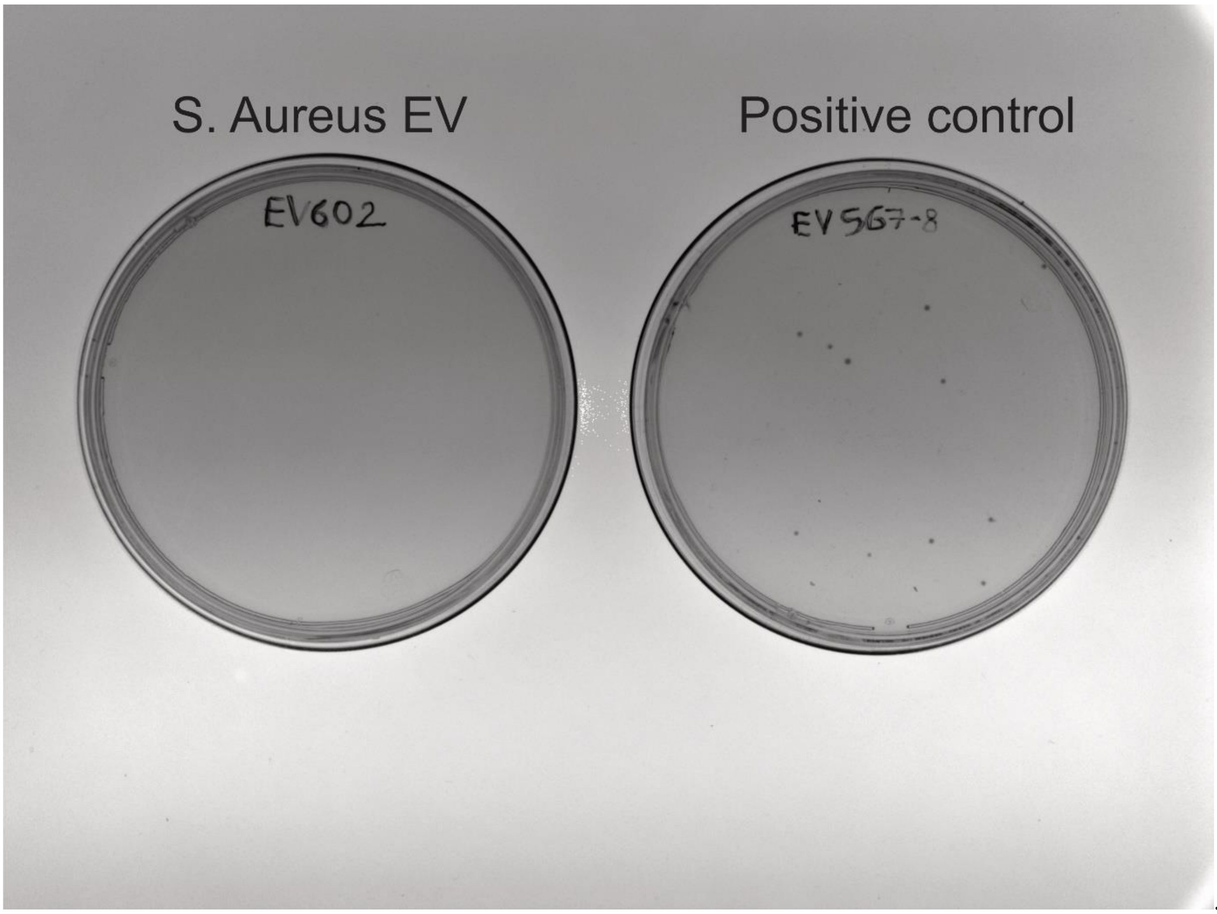

Figure S1. Luria-Bertani (LB) agar plates showing the sterility of *S. aureus* M5512VL EV pellet compared to a pellet of EVs extracted outside the sterile hood.

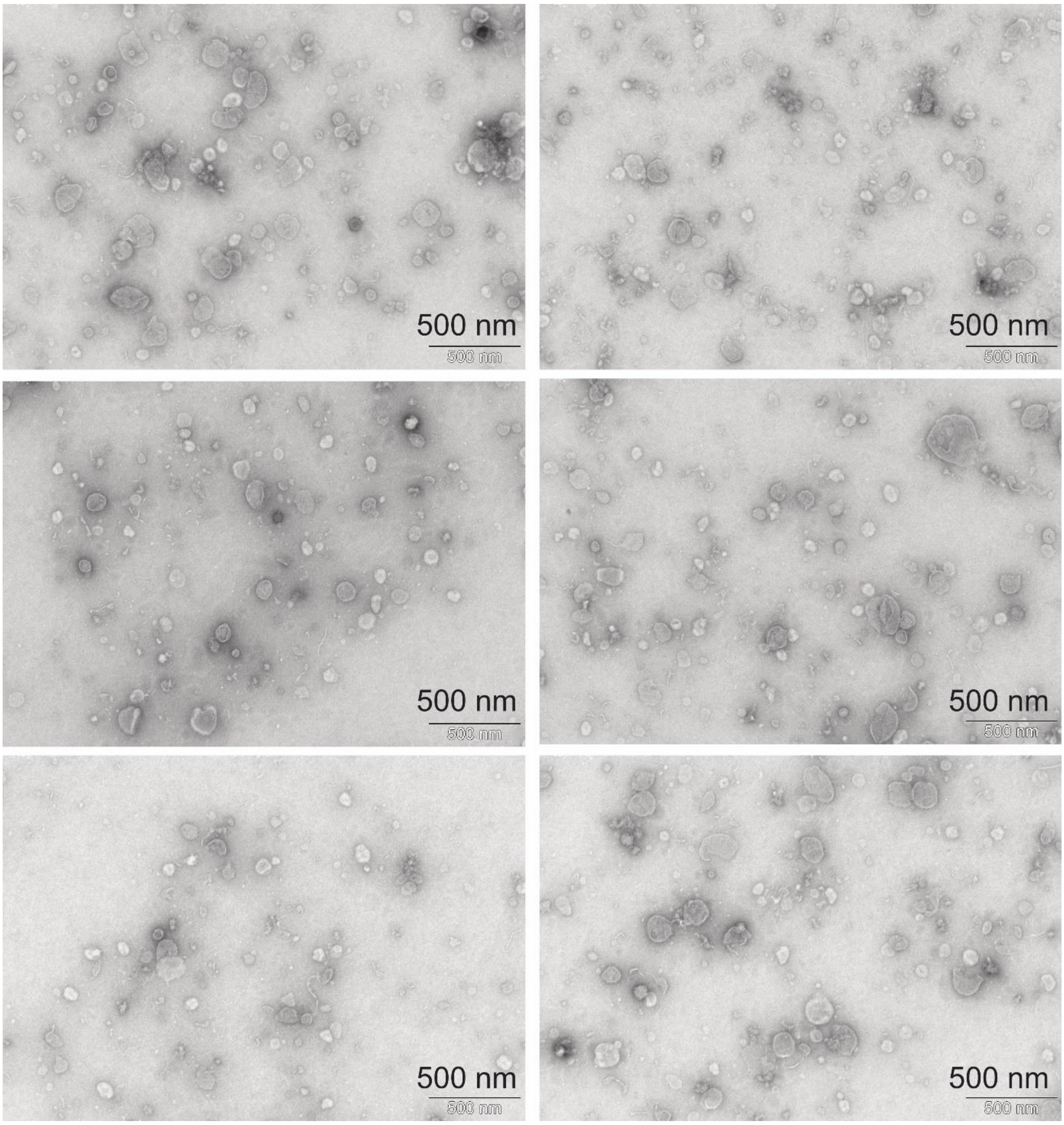

Figure S2. Transmission electron microscopy (TEM) observations of isolated EVs from *S. aureus* Mastidis.

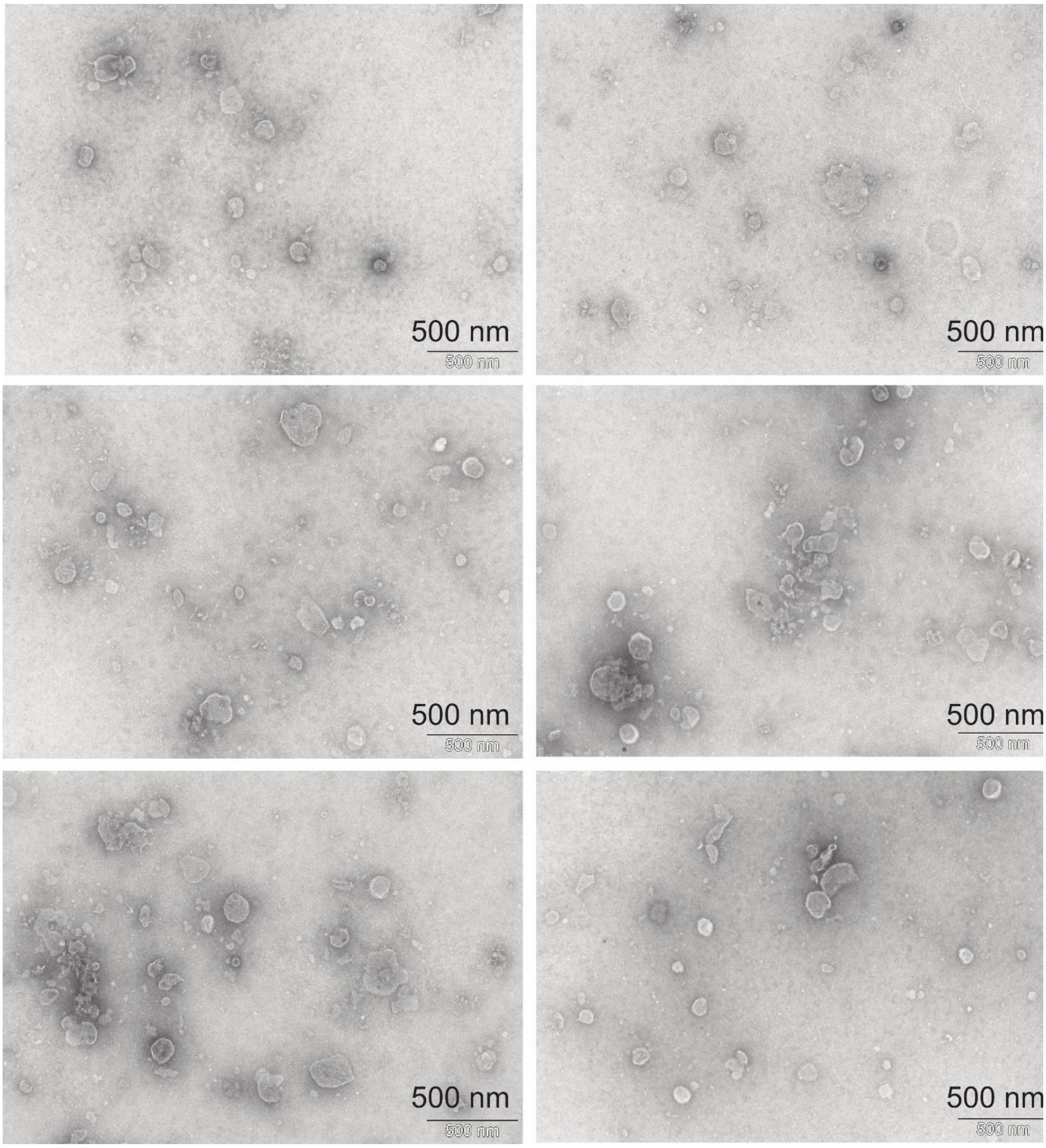

Figure S3. Transmission electron microscopy (TEM) observations of isolated EVs from *S. aureus* M5512VL.

Original Western blot Figure 2b

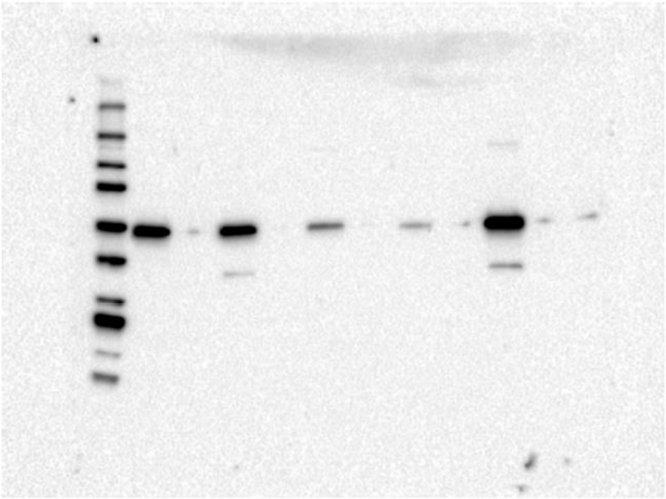

Original Western blot Figure 2c

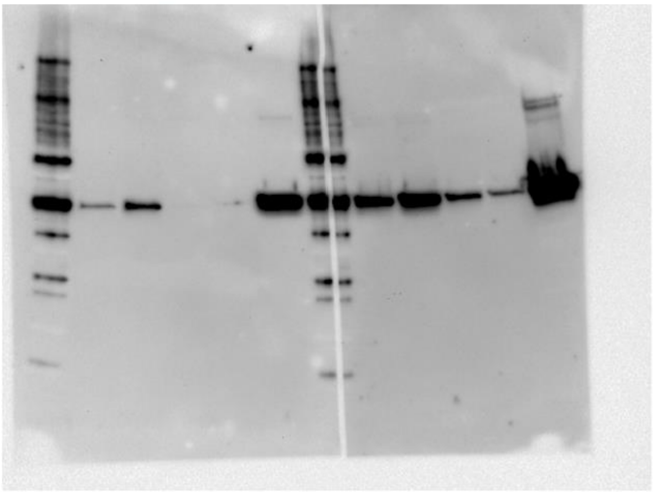

Figure S4. Original Westren blot images.

Table S1. Sequences of forward and reverse primers used for RT-qPCR analysis.

Gene		Sequence	Amplicon size (bp)
ACTB			
	Forward	GATCTGGCACCACACCTTCT	174
	Reverse	AGAGACAGCACAGCCTGGAT	
GAPDH			
	Forward	GGTCACCAGGGCTGCTTTTA	222
	Reverse	CCAGCATCACCCCACTTGAT	
IL-6			
	Forward	GCGCATGGTCGACAAAATCT	159
	Reverse	CAAATCGCCTGATTGAACCCA	
LTF			
	Forward	GATGGTGGCATGGTGTTTGA	136
	Reverse	AAGTTGCTGCCCTTCTTCAC	
NF-κB			
	Forward	ATCTGAGCATTGTGCGACTG	131
	Reverse	CTTCAGGTTTGAGGCTCCAG	
TLR2			
	Forward	CCATGTCTGGAGAGGGTGTT	140
	Reverse	GGGGACACAAAACAGCACTT	
TLR4			
	Forward	GACCCTTGCGTACAGGTTGT	103
	Reverse	GGTCCAGCATCTTGGTTGAT	
TNF-α			
	Forward	GCCCTCTGGTTCAAACACTC	193
	Reverse	AGATGAGGTAAAGCCCGTCA	
